# The implications of 18F-FDG PET for the diagnosis of endoprosthetic loosening and infection in hip and knee arthroplasty: Results from a prospective, blinded study

**DOI:** 10.1186/1471-2474-7-20

**Published:** 2006-03-03

**Authors:** K-St Delank, M Schmidt, JW-P Michael, M Dietlein, H Schicha, P Eysel

**Affiliations:** 1 Department of Orthopaedic Surgery Cologne University, Joseph-Stelzmann Straße 9, 50931 KOELN, Germany; 2 Department of Nuclear Medicine, University of Cologne, Kerpener Straße 62, 50937 KOELN, Germany; 3 Department of Orthopaedic Surgery Cologne University, Joseph-Stelzmann Straße 9, 50931 KOELN, Germany; 4 Department of Nuclear Medicine, University of Cologne, Kerpener Straße 62, 50937 KOELN, Germany; 5 Department of Nuclear Medicine, University of Cologne, Kerpener Straße 62, 50937 KOELN, Germany; 6 Department of Orthopaedic Surgery Cologne University, Joseph-Stelzmann Straße 9, 50931 KOELN, Germany

## Abstract

**Background:**

The most frequent complications of joint arthroplasty are septic or aseptic loosening of endoprostheses. Preoperative differentiation is essential, since very different treatment methods result from the diagnoses. The aim of the current study was to evaluate the clinical value of ^18^F-Fluoro-deoxyglucose positron emission tomography (^18^F-FDG PET) as a diagnostic modality for inflammation and loosening in hip and knee joint prostheses.

**Methods:**

^18^F-FDG-PET examinations and multiphase bone scan were performed on hip and knee endoprostheses in 27 patients prior to revision surgical procedures planned for prosthetic loosening. Intact prostheses were found at the opposite site in some patients so that additional 9 joints could be examined with the field of view of ^18^F-FDG PET. Verification and valuation of the PET and scintigraphic image findings were conducted by comparing them with information combined from intraoperative findings, histopathology, and microbiological investigations.

**Results:**

Evidence of loosening was correctly determined in 76.4% of cases using ^18^F-FDG-PET, and in 75% of cases using bone scan. The detection of periprosthetic inflammation using ^18^F-FDG-PET had a sensitivity of 100% for septic cases and of 45.5% in cases of increased abrasion and aseptic foreign-body reactions. However, reliable differentiation between abrasion-induced and bacterial-caused inflammation was not possible using ^18^F-FDG-PET.

**Conclusion:**

^18^F-Fluoro-deoxyglucose positron emission tomography (^18^F-FDG-PET) allows reliable prediction of peri-prosthetic septical inflammatory tissue reactions. Because of the high sensitivity of this method, a negative PET result in the setting of a diagnostically unclear situation eliminates the need for revision surgery. In contrast, a positive PET result gives no clear differentiation regarding the cause of inflammation.

## Background

In cases there is presumption of prosthetic loosening based on clinical and/or radiological evidence, prosthetic anchoring must be assessed with supplementary diagnostic examinations. In addition, prior to prosthetic revisioning it should be clarified whether the loosening is secondary to septic or aseptic causes. Particularly with regards to the operative consequences and the associated burden on patients as well as the cost of treatment, it is also desirable to categorize the intensity and extent of inflammation. Differentiation between an infection and a wear debris caused by foreign body reaction should be aimed for. In many cases, presently available investigative methods cannot satisfactorily evaluate the situation, and therefore new, more effective procedures need to be found [1].

^18^F-Fluoro-deoxyglucose positron emission tomography (^18^F-FDG PET) can be used to depict an increased glucose metabolism at the cellular level. ^18^F-FDG PET is an established method used for tumor and cardiac diagnosis as well as in neuro-nuclear medicine. It has also been determined that this glucose analogon markedly accumulates in inflammatory tissue. For this reason, ^18^F-FDG PET investigations (among others) are used for diagnosis of osteomyelitis. Since the first works of De Winter et al. [2] and van Acker et al. [3] it has been recognized that inflamed periprosthetic tissue shows ^18^F-FDG accumulation.

Relevant studies considering the semiquantitative parameter "standardized uptake value" (SUV) have shown high sensitivity of ^18^F-FDG PET with limited or unclear specificity. This problem stems from the fact that any process with increased cellular activity and therefore increased glucose utilization will eventually lead to enriched ^18^F-FDG uptake. As a result, differentiation between septic and aseptic inflammation appears to be even more difficult. However, the description of specific uptake patterns made by Reinartz et al. [4] and Cremerius et al. [5] promises possible qualitative assessment of the prostheses.

This current prospective, blinded study was carried out to define the role of ^18^F-FDG PET as a diagnostic tool for cases of hip and knee endoprosthetic loosening.

## Methods

27 patients (aged 45–82 years) were scheduled for revision surgery of total knee (n = 5) or hip (n = 22) endoprostheses on the basis of clinical, radiological, and bone scan findings indicating loosening. Two total knee prostheses were fixed without cement. 10 of the acetabular cups, and 12 of femoral shafts were implanted cementless. The remaining prostheses were fixed with cement. Surgery took place between 9/02 and 12/04 after an average time of 8.9 years (0.8–19.4 years) after primary implantation.

Permission was granted by the Ethics Commission of the Medical Faculty of the University of Cologne (study number 03 215) to carry out an ^18^F-FDG positron emission tomography on each patient preoperatively based on clinical judgement. A signed declaration of consent of each patient was presence before the investigation.

**Figure 1:**
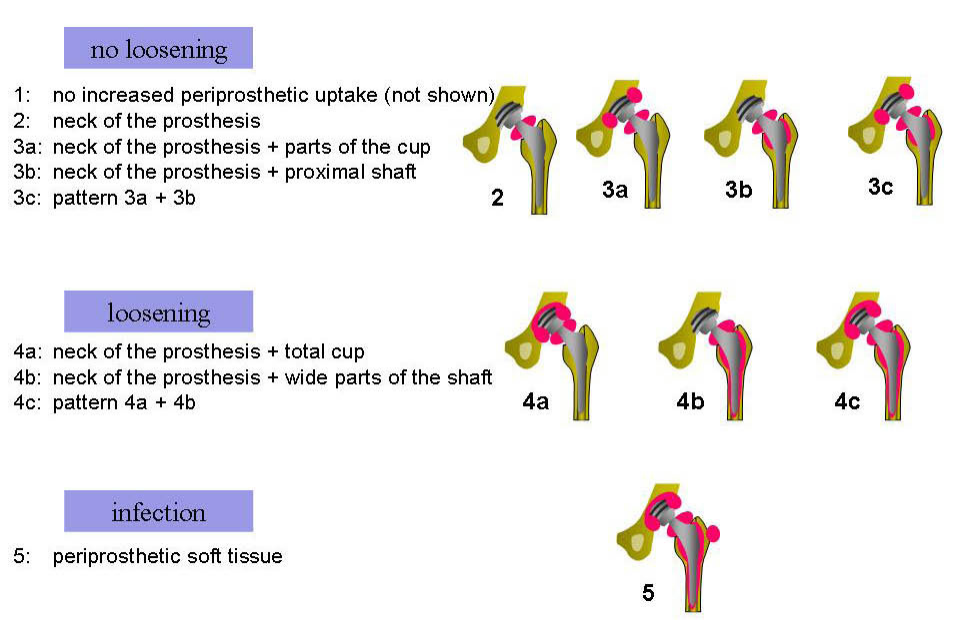
Qualitative visual analysis of tracer distribution according to the classification system of Reinartz et al. [11]

PET images were obtained with a Siemens ECAT EXACT HR scanner (Siemens Medical Systems) with an in-plane resolution of 5 mm full width half maximum in the center of the field of vision [6]. All patients fasted for at least 6 hours prior to scanning. Blood glucose levels were checked prior to ^18^F-FDG injection. Approximately 60 minutes after injection of approximately 370 MBq (10 mCi) ^18^F-FDG [7], emission scanning began, followed by transmission scanning with three germanium-68 rotating rod sources for attenuation correction. Cycles were repeated for more bed positions usually covering the region of the hip- and knee-joints. Scatter, decay, and arc corrections were performed with ECAT 7.0 software, and images were reconstructed by means of filtered back projection. Images were viewed on a Siemens workstation that permits simultaneous viewing in all three planes with easy cross-referencing between planes (Multi Purpose Imaging Tool). PET images were visually evaluated by experienced nuclear physicians, each of whom had over 4 years experience in interpreting nuclear medicine studies. If interpretation differed, images were discussed in a plenum of nuclear medicine specialists and the majority decision was binding. Qualitative visual analysis of tracer distribution (Fig. 1) was done according to the classification system for hip arthroplasty of Reinartz et al. [4].

Maximal standard uptake values (SUV) were corrected for the injected dose and patient body weight in order to obtain the standardized uptake values as follows:

SUV=tissue radioactivity concentration (Bq/ml)decay corrected injected dose (Bq)/body weight (g)×calibration factor
 MathType@MTEF@5@5@+=feaafiart1ev1aaatCvAUfKttLearuWrP9MDH5MBPbIqV92AaeXatLxBI9gBaebbnrfifHhDYfgasaacH8akY=wiFfYdH8Gipec8Eeeu0xXdbba9frFj0=OqFfea0dXdd9vqai=hGuQ8kuc9pgc9s8qqaq=dirpe0xb9q8qiLsFr0=vr0=vr0dc8meaabaqaciaacaGaaeqabaqabeGadaaakeaacqWGtbWucqWGvbqvcqWGwbGvcqGH9aqpdaWcaaqaaiabdsha0jabdMgaPjabdohaZjabdohaZjabdwha1jabdwgaLjabbccaGiabdkhaYjabdggaHjabdsgaKjabdMgaPjabd+gaVjabdggaHjabdogaJjabdsha0jabdMgaPjabdAha2jabdMgaPjabdsha0jabdMha5jabbccaGiabdogaJjabd+gaVjabd6gaUjabdogaJjabdwgaLjabd6gaUjabdsha0jabdkhaYjabdggaHjabdsha0jabdMgaPjabd+gaVjabd6gaUjabbccaGiabcIcaOiabdkeacjabdghaXjabc+caViabd2gaTjabdYgaSjabcMcaPaqaaiabdsgaKjabdwgaLjabdogaJjabdggaHjabdMha5jabbccaGiabdogaJjabd+gaVjabdkhaYjabdkhaYjabdwgaLjabdogaJjabdsha0jabdwgaLjabdsgaKjabbccaGiabdMgaPjabd6gaUjabdQgaQjabdwgaLjabdogaJjabdsha0jabdwgaLjabdsgaKjabbccaGiabdsgaKjabd+gaVjabdohaZjabdwgaLjabbccaGiabcIcaOiabdkeacjabdghaXjabcMcaPiabc+caViabdkgaIjabd+gaVjabdsgaKjabdMha5jabbccaGiabdEha3jabdwgaLjabdMgaPjabdEgaNjabdIgaOjabdsha0jabbccaGiabcIcaOiabdEgaNjabcMcaPaaacqGHxdaTcqWGJbWycqWGHbqycqWGSbaBcqWGPbqAcqWGIbGycqWGYbGCcqWGHbqycqWG0baDcqWGPbqAcqWGVbWBcqWGUbGBcqqGGaaicqWGMbGzcqWGHbqycqWGJbWycqWG0baDcqWGVbWBcqWGYbGCaaa@BF9E@

Because a number of revised patients had bilateral prostheses in place, an extra 9 joints along with the original 27 were examined with the field of view of ^18^F-FDG-PET, resulting in a total number of 36 scanned joints. The 9 joints which were not revised showed neither clinical nor radiologic evidence of loosening or inflammation.

The interface tissue between bone and loosened prostheses was examined histologically and microbiologically. These results along with intraoperative macroscopic findings were used as a "gold standard" to compare with the results of the ^18^F-FDG-PET scans. The microbiological specimens were incubated for 14 days. The time period is sufficient to increase sensitivity of the examinations from 41% to 64% [8]. 5 of the investigated 27 prostheses showed microbiological as well as histologic evidence of septic prosthetic loosening at the time of revision.

It is well known that abrasion particles from the articular surface cause periprosthetic inflammation. For this study, the extent of abrasion was semiquantitatively classified using radiologic and intraoperative findings. Using this system, the aseptic joints were classified as 11 loosened prostheses with "increased abrasion," and 20 loosened prostheses with "no or little abrasion." The radiological measure of PE-abrasion is decentralization of the head within the cup. Distance from craniolateral and caudomedial cup to the margo of the head was measured. Halve of difference of these two distances was according to decentralization of the head. „Increased abrasion" was established in case of a distance of decentralization over 2.5 mm. All 9 prostheses which were not revised and not loosened prostheses were evaluated as "no or little abrasion". For this group of prostheses there was also no evidence of bacterial infection.

The PET investigators (M.S./M.D./H.S.) were blinded to all other findings. After the specific uptake pattern of each prostheses was determined as described above, the PET findings were evaluated for presence of loosening and determination of aseptic versus septic inflammatory reaction. The PET-based assessments were compared with the actual intraoperative findings. and classified as true positive/negative or false positive/negative.

In 28 joints (22/27 revised prostheses and 6/9 not revised prostheses), additional bone scan in triple-phase technique including SPECT was performed. 500 MBq Tc-99 m MDP were injected. Negative perfusion and blood pool but positive osseous phases were interpreted as loosening. Positive perfusion, blood pool and positive osseous phase were interpreted as infection. The bone scan-based evaluations were similarly compared with the actual intraoperative findings and classified as true positive/negative or false positive/negative.

Data were analysed using descriptive statistics expressing values as mean ± standard deviation. Groups were compared by use of Mann Whitney test and results were considered significant in case of a p-Value < 0.05.

## Results

### Loosening of prostheses

**Figure 2:**
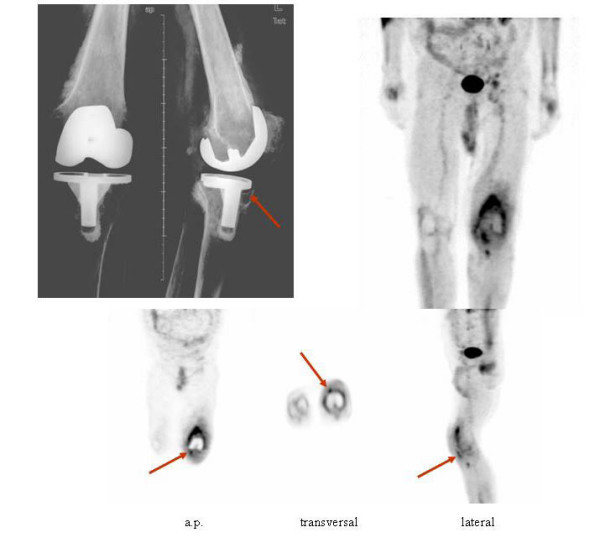
Intraoperatively confirmed loosening of the proximal and distal components. An infection with coagulase negative staphylococcus was also identified. PET was initially interpreted as synovialitis without signs of mechanical loosening. The inflamed synovium as well as the periprosthetic osteolytic areas (arrow) filled with bacteria-infected granulation tissue showed ^18^F-FDG uptake.

**Figure 3:**
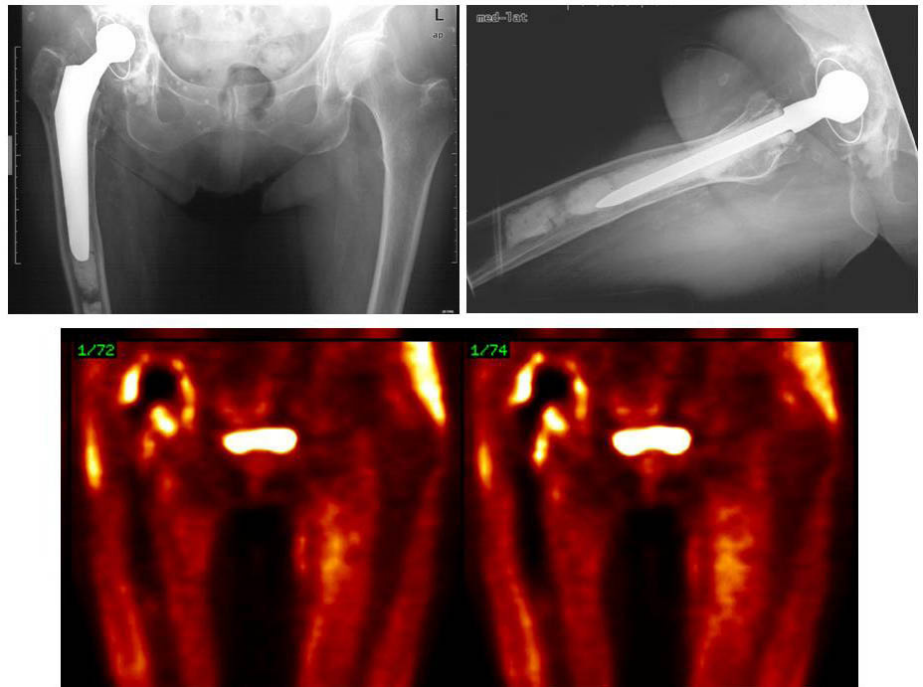
^18^F-FDG-PET showed periacetabular uptake (pattern 4a) as evidence of loosening of the cup confirmed by surgery. A bacterial infection was excluded. Loosening of the shaft within the cement bed is not adequately depicted by ^18^F-FDG-PET.

**Figure 4:**
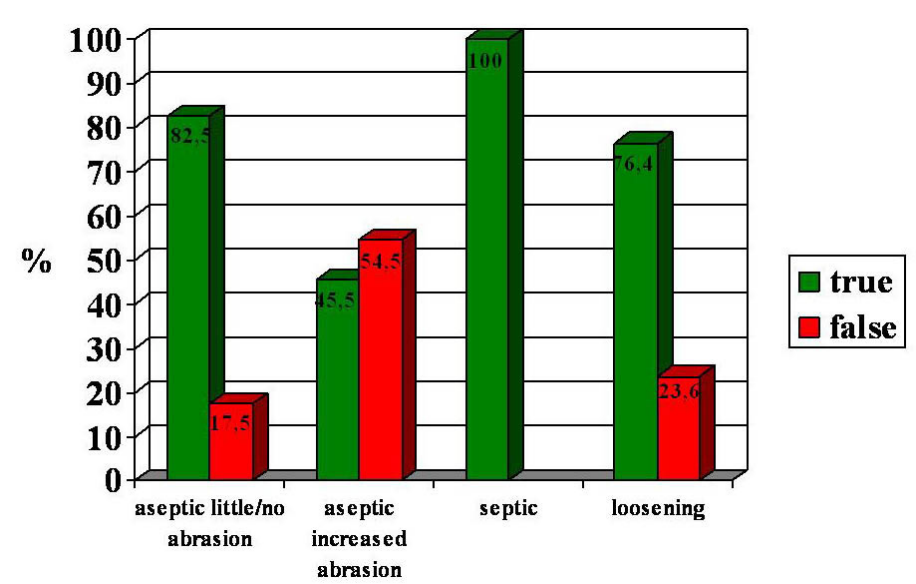
PET findings with reference to mechanical prosthetic loosening and inflammation.

Regarding the 27 revised hip and knee prostheses, intraoperative findings showed loosening of the proximal component in 16 cases and loosening of the distal component in 15 cases. A prosthetic component was classified as loosened when its anchoring released without or with minimal mechanical force asserted with few hammer blows. 83% of the loosend implants radiological evaluation showed radiolucent lines. Loosening of proximal or distal component showed the specific PET uptake patterns (Fig. 1) in the frequencies depicted in Table 1b. A specific uptake pattern distinguishing type of prosthetic loosening could not be compiled (Tab. 1). But it is conspicuous that an increased uptake in the middle of the cup (area10, DeLee and Charnley)[9] or in the distal portion of the stem (area 2–6, Gruen et al.) [10] point to a prosthetic loosening. The PET-based assessment of mechanical prosthetic loosening was correct in only 76.4% of cases (Fig. 2,3). There was no significant difference between cases identified involving the proximal versus distal components (Fig. 4).

Bone scan produced true results regarding the criteria "loosening" in 75% of cases.

### Inflammation of prostheses

In five cases, the prosthetic loosening was found in association with a microbiologically confirmed bacterial infection of the prostheses. Evidence for increased polyethylene abrasion with periarticular foreign body reactions was found in 11 cases.

The specific PET uptake patterns signaling inflammation were categorized using the classification from Reinartz et al. [4] in the frequencies depicted in Table 1a.

The semiquantitative analysis of FDG uptake (Standard Uptake Value or SUV) showed the highest values in cases of septic prostheses. In cases of increased abrasion and aseptic foreign-body reactions there were equally high values (p = 0,1004). Compared to cases classified with little or no abrasion (Table 2a) the septic group showed significantly higher values (p = 0,0209). Statistical analysis of Standard Uptake value of hip prostheses showed significant higher values for cemented fixation (Table 2b). PET-based evaluations of inflammation were correct in 82.5% (true negative) in cases of little/no abrasion and in 45.5% (true positive) in cases of increased abrasion and aseptic foreign-body reactions (Fig. 4). However, rating of true positive cases is critically dependent where the cutoff is set.

17.5% showed a false positive result for inflammation with a PET-based assessment when intraoperative results were negative. There were no septic cases designated with a false negative result.

**Figure 5:**
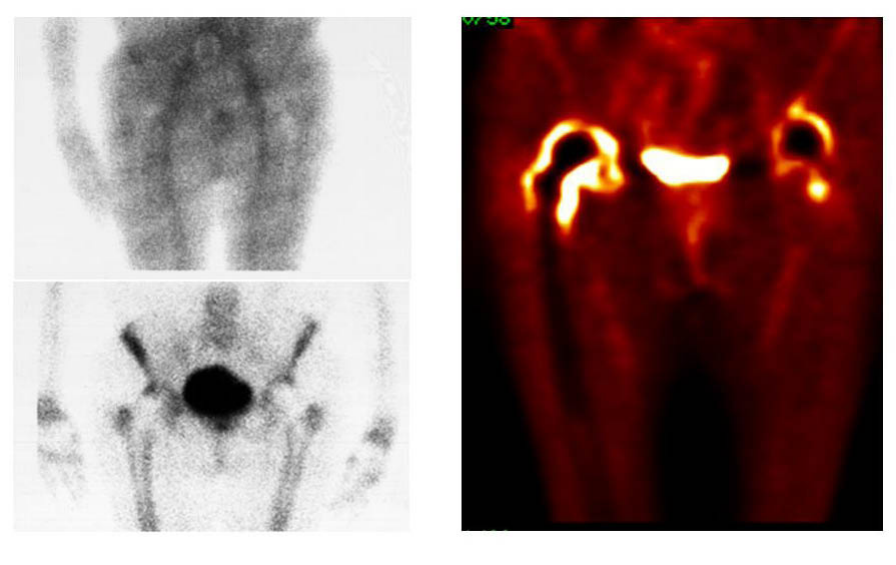
In a case with extensive abrasion-induced inflammation on the right hip prosthesis (bacterial infection was excluded) whereas bone scan in comparison to 18F-FDG-PET does not adequately demonstrate the pathology. The PET examination shows broad periprosthetic uptake as expression of synovitis.

Bone scan produced true positive results regarding the criteria "inflammation" in cases of increased abrasion and aseptic foreign-body reactions only in one case. 93.7% of inflammation were not adequately detected by bone scan (Fig. 5). One of 5 septic prostheses was not detected (false negative) by bone scan.

## Discussion

The aim of the present study was to evaluate the diagnostic accuracy and the resulting significance of ^18^F-FDG positron emission tomography for the assessment of peri-prosthetic loosening and inflammation of total joint replacements. Accuracy of this method for prediction mechanical loosening is the first question. Second is the clarification of the sensitivity and specificity with which periprosthetic inflammation is recognized and the question of whether it is possible to differentiate between aseptic (abrasion-caused) and septic inflammation using this modality. Our basic hypothesis was that cellular composition and the associated glucose utilization of the periprosthetic tissues varies enough for a difference in quantitative and/or qualitative uptake by ^18^F-FDG PET can be measured. Differentiation of cellular composition of the periprosthetic membranes is currently possible using gene expression analysis with cDNA microrays [11].

36 joints in total were investigated with ^18^F-FDG positron emissions tomography. In 27 cases the results were correlated with the gold-standard of intraoperatively-taken macroscopic, histologic, and microbiologic findings.

Incidental observations made during the established uses of ^18^F-FDG PET for tumor and cardiac diagnosis have shown that inflamed tissues utilize increased amounts of glucose analogs [12]. A high sensitivity for general musculoskeletal infections has also already been established [13].

The current study shows that with a sensitivity of 100%, ^18^F-FDG PET is a reliable diagnostic modality for the identification of periprosthetic septical reactions. In cases of a periprosthetic aseptic foreign-body reactions the inflammation is not as good represented by ^18^F-FDG PET as in septical inflammation (sensitivity 45.5%) but better than by sceletal scintigraphy (6.3%).

Overlap in uptake patterns of prostheses with "increased" or "little-no" abrasion due to histopathological different reactions [14].

Considering that septical as well as heavy aseptical inflammation lead to progressive bony destruction, a high sensitivity is desirable. Using ^18^F-FDG PET as one step of the diagnostic cascade corresponding inflammatory reactions can be identified at an early stage and the necessary operative steps taken as soon as necessary. The specificity of this modality is markedly limited in comparison to the sensitivity. Although the semiquantitative analysis of glucose analog uptake (**S**tandard **U**ptake **V**alue) did in fact show higher values for cases of septic inflammation as well as increased abrasion-induced inflammation, there was no significant difference measured (p = 0.1004). The same problem was previously identified by other research groups [2,15], and the descriptive assessment of uptake patterns was therefore introduced to improve selectivity. The uptake pattern classification for hip prostheses introduced by Reinartz [4] was used in the present study. The breakdown shows that all cases of septic inflammation showed surface-wide peri-prosthetic uptake (patterns 4a-c, and 5). However, it also showed that aseptic, abrasion-caused inflammation could have similar patterns (4a and 4b). This phenomenon is understandable; ^18^F-Fluoro-deoxyglucose is a nonspecific radiopharmaceutical agent that does not lead to direct germ identification. Therefore, sufficient detection of inflammatory reactions is possible without differentiation between septic and aseptic etiologies. Beside this specific uptake patterns of hip und knee joints differ. Especially synovialitis of the knee is more worse. Consequently a wide uptake often is observed even without infection. Inflammation along the bone-prostheses interface, from which mechanical prosthetic loosening can develop, can be identified with ^18^F-FDG PET.

In contrast, however, the mechanical loosening itself cannot be sufficiently identified using this modality. Of 31 cases confirmed as loosened in the operating room, only 15 (76.4%) were correctly identified as "true positives" with PET. The PET scanning was particularly unsuitable to identify cases of mechanical loosening occurring between the cement bed and the prosthesis, since the lack of cellular elements in this area precludes glucose analog uptake. Despite the decreased specificity of the method, ^18^F-FDG PET has an important clinical meaning in the evaluation of a painful endoprostheses. On the one hand, the presumption of septic prosthetic loosening can be ruled out with a negative PET result, since the sensitivity of the test is high. In such a case the substantial financial and operative consequences of a septic prosthetic revision can be avoided, and finally overall costs would be reduced due to PET.

On the other hand, a prominent abrasion-mediated inflammatory reaction could be identified using ^18^F-FDG PET well before such a reaction would be recognizable radiologically or scintigraphically. The abrasion material initially accumulates in the prosthetic neck region and leads to a marked inflammation with resulting osteolysis. The osteolytic reaction progresses centripetally and eventually leads to prosthetic loosening. Using ^18^F-FDG PET, this foreign-body reaction can be shown with high sensitivity. The test therefore makes it possible to decide whether close radiologic observation is necessary or whether, in certain cases with advanced osteolytic changes, early revision should be contemplated.

## Conlusion

^18^F-Fluoro-deoxyglucose positron emission tomography (^18^F-FDG-PET) is a reliable method with a high sensitivity for detection a peri-prosthetic inflammation. Differentiation between septic and aseptic inflammation is not possible. However in case of a suspected septic inflammation a negative PET result eliminates the need for revision surgery.

## Competing interests

The author(s) declare that they have no competing interests.

## Authors' contributions

KSD: Idea, conception and design of the study.

Acquisition of data.

Evaluation of data.

Preparing the manuscript.

Revision of the manuscript and final approval.

MS: Idea, conception and design of the study.

Acquisition of data.

Evaluation of data.

Statistical analysis.

Revision of the manuscript and final approval.

JWPM: Revision of the manuscript and final approval.

MD: Acquisition of data.

Revision of the manuscript and final approval.

HS: Conception and design of the study.

Revision of the manuscript and final approval.

PE: Conception and design of the study.

Revision of the manuscript and final approval.

## Acknowledgements

This study was performed with approval of the local animal care committee.

It was supported by all members of staff from the Department of Orthopaedic surgery and Nuclear medicine, University of Cologne, Germany.

## Pre-publication history

The pre-publication history for this paper can be accessed here:



## Figures and Tables

**Table 1:** Frequency of specific PET uptake patterns according to the classification system of Reinartz [11] a) Applied to inflammatory situationb) Applied to loosening localization

		n	1	2	3a	3b	3c	4a	4b	4c	5
a)	Septic	5						1	1	1	2
	Aseptic with increased abrasion	11		1	1		3	4	2		
	Aseptic with little/no abrasion	20		5	6	1	5	3			

b)	Loosened proximal component	16		1	2		4	7	1	1	
	Loosened distal component	15			3		4	2	3	1	2
	No loosened component	23		3	3	2	6	5	2	0	2

**Table 2:** Standard uptake values (SUV) (sign. Differences <0,05) differentiated bya) inflammatory situation andb) type of fixation

**a)**				
	
		Septic Prostheses (n = 5)	Aseptic Prostheses with increased abrasion (n = 11)	Aseptic Prostheses with little/no abrasion (n = 20)
Average		4.7	3.7 (p = 0.1004)	3.4 (p = 0.0209)
Standard Deviation		1.0	1.6	1.3

b)				
	
		Cemented hip prostheses (n = 17)	Cementless hip prostheses (n = 14)	p-value

Average	shaft	4.2	3.0	0.0390
Standard Deviation		1.6	1.1	
Average	cup	4.17	3.17	0.0753
Standard Deviation		1.7	1.2	
